# Sonological appearance of idiopathic arterial calcification in fetus: A rare case

**DOI:** 10.4103/0971-3026.54876

**Published:** 2009-08

**Authors:** BR Nagaraj, Prakash Jain, Deepu Alex Thomas, M Raghu

**Affiliations:** Department of Radio Diagnosis, Bangalore Medical College and Research Institute, Bangalore, India

**Keywords:** Echogenic aorta, fetal hydrops, idiopathic arterial calcification

## Abstract

Idiopathic arterial calcification (IAC) is a rare disease characterized by extensive arterial wall calcification. This condition is almost always fatal. A total of 162 cases have been reported to date, with most cases diagnosed postnatally and less than 13 cases having been suspected antenatally. This case report describes a case of IAC detected antenatally with USG at 28 weeks' gestation.

## Introduction

Idiopathic arterial calcification (IAC) is a rare cause of arterial calcification[1] and results in death in early life. Idiopathic arterial calcification presents with varied clinical symptoms in infancy and childhood.[2] Death is due to refractory hypertension and cardiac failure.[3] A total of 162 cases[4,5] have been reported in the literature, with most cases diagnosed postnatally by autopsy and less than 13 cases[6] detected antenatally.

USG is a sensitive and accepted method for the prenatal detection of IAC.[7]

## Case Report

An unbooked, 25-year-old, consanguineously married woman with 7 months' amenorrhea was referred for routine obstetric USG. Her parity index was gravida 3, para 2, living 1 and dead 1. Her first child was healthy and the second baby had died 1 week after birth; the cause of neonatal death was not known. Her hemoglobin was 9 gm/100 ml; she was Rh positive. The TORCH test was negative. Maternal serum calcium, phosphorus and alkaline phosphatase levels were within normal limits.

**Figure 1:**
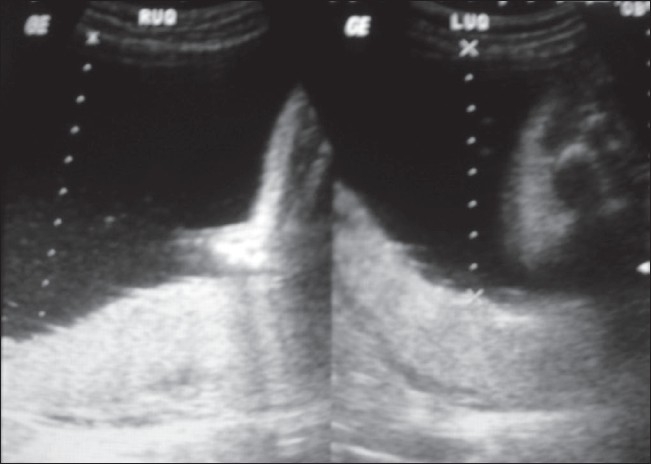
Abdominal USG of the uterus of the pregnant woman shows increased amniotic fluid (cursor)

**Figure 2:**
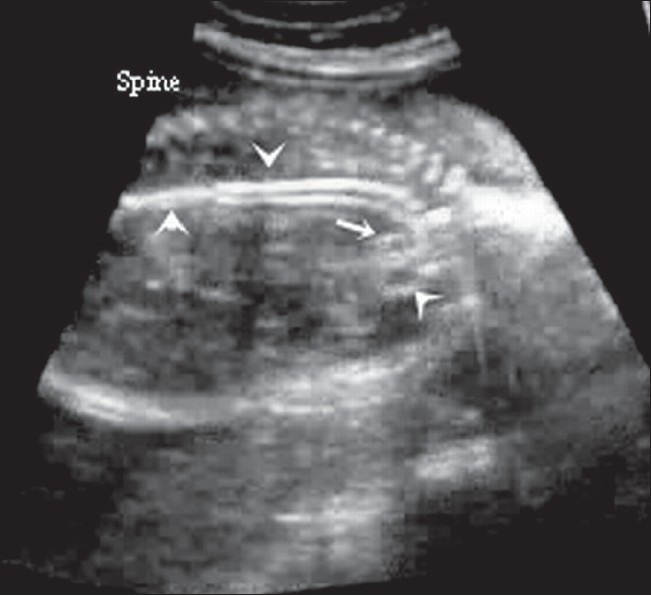
Sagittal USG of the fetal chest shows an echogenic aorta (arrowhead). The pulmonary artery is well seen (small arrow)

**Figure 3:**
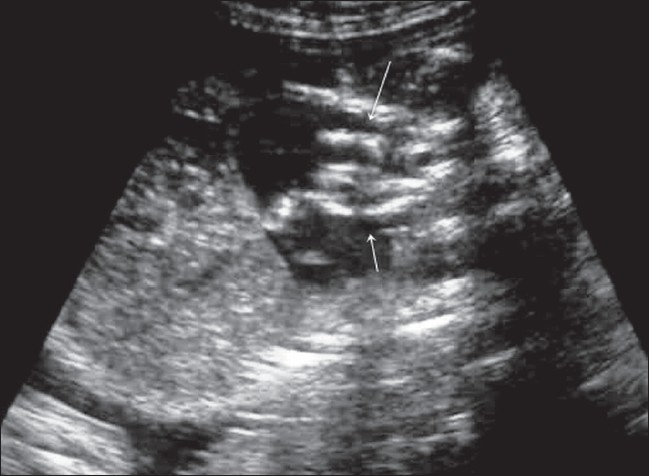
Oblique USG through the fetal chest shows echogenic outflow tracts (arrows)

**Figure 4:**
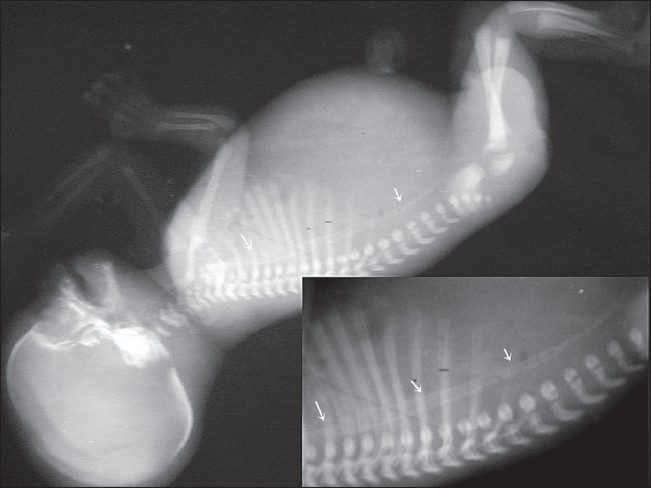
Lateral infantogram radiograph shows calcification of the aorta and iliac vessels (arrows)

**Figure 5 (A, B):**
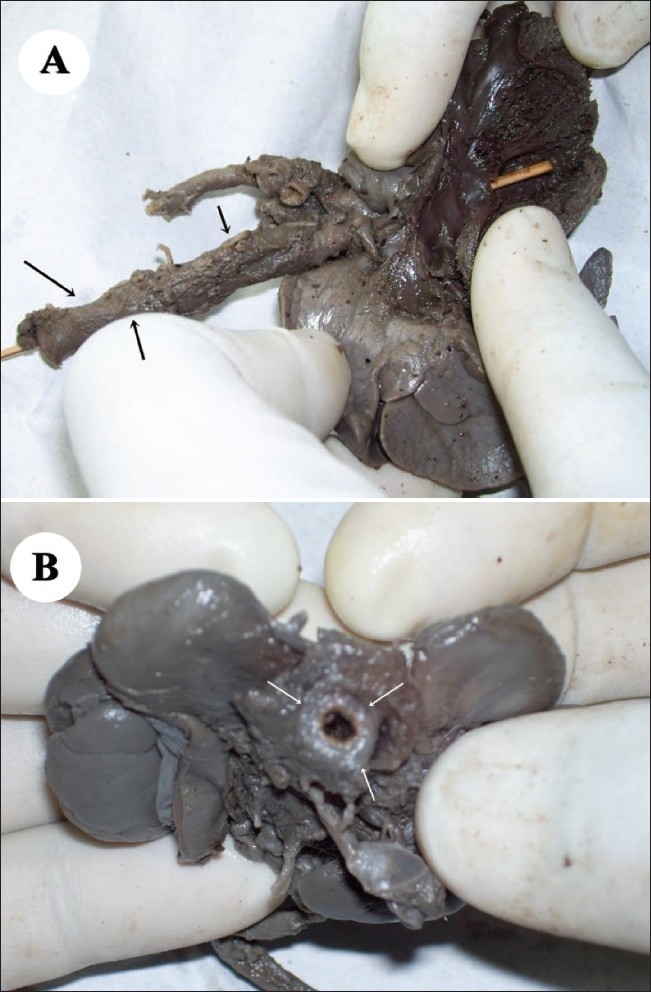
Gross specimen shows a thick, cord-like aorta (arrows) in profile (A) and en face (B)

Antenatal USG (Logiq 200, Wipro GE, Bangalore, India) revealed a single live fetus in cephalic presentation at 28 weeks of gestation. Important findings included severe polyhydramnios (amniotic fluid index: 34) [Figure 1], a highly echogenic aortic wall [Figure 2], echogenic cardiac outflow tracts [Figure 3], pericardial effusion, a large atrial septal defect, giving the appearance of a single atrium, bilateral pleural effusion, ascites, subcutaneous edema and echogenic kidneys. The rest of the organs appeared normal. Early grade III maturity of the placenta was noted. On the basis of the gray-scale findings, a diagnosis of IAC was made and fetal echo and follow-up scan were suggested. Follow-up scan after 1 week showed intrauterine fetal demise. The dead fetus was delivered vaginally. Radiographic examination confirmed aortic calcification [Figure 4]. Autopsy of the fetus confirmed the other findings such as fetal hydrops, thickened cord-like aorta [Figure 5 AB] and large atrial septal defect. Histopathological examination showed calcification of the tunica intima and media. Sections from the kidneys, spleen, liver and thymus showed calcific deposits and parenchymal psammoma bodies.

## Discussion

Idiopathic arterial calcification was first reported in 1901 by Bryant and White.[8] Until now, 162 cases have been reported. The exact etiology of IAC is not known. An autosomal recessive inheritance pattern has been described. Recently, a genetic etiology was postulated: a homozygous or compound heterozygous nonsense mutation for the ecto-nucleotide pyrophosphatase/phosphodiesterase 1 (*ENPP1*) gene on chromosome 6q.[9] The ENPP1 gene regulates extracellular inorganic pyrophosphate (PPi) level, and PPi regulates the matrix calcification. Thus, the ENPP1 mutation results in extracellular matrix calcification.[10] Histologically, this is evident as intimal fibrous proliferation and calcium hydroxyapatite deposition in the intimal elastic lamina of the arteries.[4,11] Extensive arterial calcification results in refractory hypertension and cardiac failure, and involvement of the coronary arteries causes myocardial ischemia.[3,4,5] In the antenatal period, IAC presents with polyhydramnios, fetal hydrops or still birth. Most cases are diagnosed in infancy and, therefore, in some published reviews the condition is referred to as idiopathic arterial calcification (IAC) of infancy.[2] Due to its rarity, the varied clinical presentation, and the rapid clinical worsening, most cases are diagnosed only at autopsy.[4,12] Very few cases have been diagnosed antenatally on the basis of USG findings.

Common findings described on antenatal USG include echogenic aorta and outflow tracts, polyhydramnios, pericardial effusion and hydrops. Of these, echogenic aorta and outflow tracts are consistently seen [Table 1]. Our case showed all the above findings and, in addition, there were pleural effusions, ascites, edematous skin/subcutaneous tissue and a large atrial septal defect. All antenatally detected cases in the literature have been reported in the late second and thirdth trimesters, with the earliest detected case being at 26 weeks; our case was detected at 28 weeks of gestation [Table 1].

**Table 1:** Reported antenatal ultrasound findings in idiopathic arterial calcification

No. of cases	Report and year	Gestational age in weeks	Sonographic findings			
			
			Polyhydramnios	Echogenic aorta	Pericardial effusion	Hydrops fetalis
1	Rosenbaum *et al*. 1986[2]	32	NM	P	NM	P
2	Stuart *et al*. 1990[13]	33	NM	P	P	NM
3 and 4	Spear *et al*. 1990[14]	Twin I 32	P	P	NM	P
		Twin II 31.5	NM	P	P	P
5	Samon *et al*. 1995[15]	28	P	P	P	NM
6 and 7	Eronen *et al*. 2001[3]	Twin I 29	P	NM	P	P
		Twin II 30	P	P	P	P
8	Levine *et al*. 2001[16]	32	P	P	P	NM
9	Nagaret *et al*. 2003[6]	27	P	P	P	P
10	Ciana *et al*. 2006[17]	26	NM	P	NM	NM

P - Present in the reported case, NM - Not mentioned in the reported case

In this case, the parents were consanguineously married; the first child was normal and the second child had died during the early neonatal period. This history supports a genetic etiology. Confirmation of the USG findings was done by radiographic and postmortem examination.

Antenatal diagnosis of IAC can be made in the presence of an echogenic aorta, echogenic outflow tracts, polyhydramnios, pericardial effusion and fetal hydrops. A family history of IAC or polyhydramnios, with a previous perinatal death, should prompt antenatal screening for IAC; early detection of cases helps in counseling of the parents. An echocardiogram and genetic screening for ENPP1 mutation may be indicated in suspected cases.[4]

Until recently, IAC was considered a fatal condition. However, in a recent report, postnatal therapy with nitrogen-containing bisphosphonates such as low-dose disodium pamidronate, has shown rapid resolution of the arterial calcifications and better survival of the affected patients.[18]

## References

[1] Bird T (1974). Idiopathic arterial calcification in infancy. Arch Dis Child.

[2] Rosenbaum DM, Blumhagen JD (1986). Sonographic recognition of idiopathic arterial calcification of infancy. AJR Am J Roentgenol.

[3] Eronen M, Pohjavuori M, Heikkila P (2001). Fatal outcome of 2 siblings with idiopathic arterial calcification of infancy diagnosed in utero. Pediatr Cardiol.

[4] Glatz AC, Pawel BR, Hsu DT, Weinberg P, Chrisant MR (2006). Idiopathic infantile arterial calcification: Two case reports, a review of the literature and a role for cardiac transplantation. Pediatr Transplant.

[5] Chong C, Hutchins G (2007). Idiopathic Infantile Arterial Calcification: The Spectrum of Clinical Presentations. Pediatr Dev Pathol.

[6] Nagar AM, Hanchate V, Tandon A, Thakkar H, Chaubal NG (2003). Antenatal Detection of Idiopathic Arterial Calcification With Hydrops Fetalis. J Ultrasound Med.

[7] Bellah RD, Zawodniak L, Librizzi RJ, Harris MC (1992). Idiopathic arterial calcification of infancy: prenatal and postnatal effects of therapy in an infant. J Pediatr.

[8] Bryant JH, White WA (1901). A case of calcification of the arteries and obliterative endarteritis associated with hydronephrosis in a child aged 6 months. Guys Hosp Rep.

[9] Rutsch F, Ruf N, Vaingankar S, Toliat MR, Suk A, Höhne W (2003). Mutations in ENPP1 are associated with ‘idiopathic’ infantile arterial calcification. Nat Genet.

[10] Numakura C, Yamada M, Ariyasu D, Maesaka A, Kobayashi H, Nishimura G (2006). Genetic and enzymatic analysis for two Japanese patients with idiopathic infantile arterial calcification. J Bone Miner Metab.

[11] Pashankar D, Moore L (1997). Test and teach. Number eighty three: Part 1. Idiopathic arterial calcification of infancy. Pathology.

[12] Saigal G, Azouz EM (2004). The spectrum of radiologic findings in idiopathic arterial calcification of infancy: pictorial essay. Can Assoc Radiol J.

[13] Stuart G, Wren C, Bain H (1990). Idiopathic infantile arterial calcification in two siblings: failure of treatment with diphosphonate. Br Heart J.

[14] Spear R, Mack LA, Benedetti TJ, Cole RE (1990). Idiopathic infantile arterial calcification: in utero diagnosis. J Ultrasound Med.

[15] Samon LM, Ash KM, Murdison KA (1995). Aorto-pulmonary calcification: an unusual manifestation of idiopathic calcification of infancy evident antenatally. Obstet Gynecol.

[16] Levine JC, Campbell J, Nadel A (2001). Prenatal diagnosis of idiopathic infantile arterial calcification. Circulation.

[17] Ciana G, Trappan A, Bembi B, Benettoni A, Maso G, Zennaro F (2006). Generalized arterial calcification of infancy: Two siblings with prolonged survival. Eur J Pediatr.

[18] Ramjan KA, Roscioli T, Rutsch F, Sillence D, Munns CF (2009). Generalized arterial calcification of infancy: Treatment with bisphosphonates. Nat Clin Pract Endocrinol Metab.

